# Association of multimodal analgesic protocol with postpartum depression incidence and sleep quality in high-risk parturients

**DOI:** 10.3389/fmed.2026.1828838

**Published:** 2026-05-20

**Authors:** Hui Zhang, Yan an Jiang, Huajun Fu, Lin Zhang, Gang Zhang

**Affiliations:** Department of Anesthesiology, Shaanxi Provincial People’s Hospital, Xi’an, Shaanxi, China

**Keywords:** cesarean delivery, dexmedetomidine, esketamine, high-risk parturients, multimodal analgesia, postpartum depression, sleep quality

## Abstract

**Background:**

Postpartum depression (PPD) affects 10–20% of women after childbirth, with incidence reaching 30–50% in high-risk populations. Acute postoperative pain and sleep disturbance represent modifiable risk factors. This study evaluated whether a multimodal analgesic protocol combining dexmedetomidine and esketamine is associated with reduced PPD incidence in high-risk parturients undergoing cesarean delivery.

**Methods:**

This single-center, retrospective cohort study included 82 high-risk parturients who received intraoperative esketamine (0.25 mg/kg) followed by postoperative dexmedetomidine-esketamine patient-controlled analgesia for 48 h between January 2023 and December 2024. Historical controls (*n* = 79) from 2022 received standard sufentanil-based analgesia. The primary outcome was PPD incidence at 6 weeks (Edinburgh Postnatal Depression Scale >10).

**Results:**

PPD incidence was significantly lower in the intervention group versus controls (14.6% vs. 29.1%; relative risk 0.50, 95% confidence interval 0.27–0.93; *p* = 0.028; number needed to treat 6.9). Edinburgh Postnatal Depression Scale (EPDS) score decreased more in the intervention group (−2.8 ± 3.4 vs. +0.6 ± 4.1; *p* < 0.001). Sleep quality at postoperative day 7 was better (Pittsburgh Sleep Quality Index 6.2 ± 2.8 vs. 8.9 ± 3.1; *p* < 0.001). Opioid consumption decreased by 21.2% (*p* < 0.001). Exploratory biomarker assessment in a subset suggested lower interleukin-6 and higher brain-derived neurotrophic factor (both *p* < 0.001), though these data were non-systematic. Psychotomimetic effects occurred in 8.5%, all transient. No significant differences in cardiovascular or respiratory adverse events were observed.

**Conclusion:**

A multimodal analgesic protocol incorporating dexmedetomidine-esketamine multimodal analgesic protocol was associated with lower postpartum depression incidence, better sleep quality, and acceptable safety in high-risk parturients. Randomized trials are needed to establish causality.

## 1 Introduction

Postpartum depression (PPD) affects 10–20% of women globally and up to 30–50% of those with risk factors (1, 2), with consequences extending to impaired mother-infant bonding, disrupted breastfeeding, and adverse offspring neurodevelopment (3, 4). Despite this substantial public health burden, effective preventive strategies remain limited, particularly for high-risk populations (5).

The peripartum period represents a critical window for intervention, as acute postoperative pain and sleep disturbance are established modifiable risk factors for PPD (6). Cesarean delivery—which accounts for 30–40% of births worldwide—further increases susceptibility through higher pain intensity and pronounced sleep fragmentation compared with vaginal delivery (7, 8). Furthermore, surgical trauma triggers inflammatory responses and brain-derived neurotrophic factor (BDNF) dysregulation that may independently precipitate depressive symptoms through neuroimmune mechanisms (9, 10). These converging pathways suggest that optimized perioperative care targeting pain, sleep, and inflammation could mitigate PPD risk following cesarean delivery.

Against this background, esketamine—the S-enantiomer of ketamine—has emerged as a rapidly acting antidepressant through NMDA receptor antagonism, enhancement of BDNF signaling, and suppression of pro-inflammatory cytokines (11). A recent randomized trial demonstrated that intraoperative esketamine (0.25 mg/kg) significantly reduced PPD incidence at 6 weeks postpartum (12). However, whether combining esketamine with complementary agents in a multimodal approach is associated with incremental benefits remains incompletely explored.

Dexmedetomidine, a highly selective α2-adrenergic receptor agonist, offers pharmacological properties that may co-vary with esketamine’s effects. Its analgesic-sparing effects reduce opioid requirements, while its ability to promote natural sleep architecture through activation of endogenous sleep pathways addresses the sleep disturbances that frequently accompany PPD. (13, 14) Meta-analytic evidence confirms that perioperative dexmedetomidine significantly improves postoperative sleep quality across diverse surgical populations (15), and its intrinsic anti-inflammatory and anxiolytic properties may further contribute to psychological well-being during the vulnerable postpartum period (16, 17).

This retrospective cohort study was designed to assess the clinical effectiveness of a standardized multimodal analgesic protocol incorporating intraoperative esketamine followed by postoperative dexmedetomidine-esketamine-based analgesia in high-risk parturients undergoing cesarean delivery. We hypothesized that this intervention would be associated with reduced PPD incidence and severity, improved early postoperative sleep quality, and acceptable safety profiles compared with historical controls receiving standard opioid-based analgesia.

## 2 Methods

### 2.1 Study design and setting

This single-center, retrospective cohort study with historical controls was conducted at the Department of Anesthesiology and Obstetrics of our hospital. This study was approved by the Institutional Ethics Committee of Shaanxi Provincial People’s Hospital (No. 202411254, November 2024) with waiver of informed consent for de-identified data. The multimodal analgesic protocol was implemented as a departmental quality improvement initiative (January 2023) following Anesthesiology Quality Committee approval, Pharmacy Department review, and Medical Affairs filing. Patients provided standard clinical consent for anesthesia and postoperative analgesia.

To address potential temporal confounding, we verified through institutional quality records that no systematic changes occurred in surgical techniques, anesthesia protocols, postpartum nursing standards, or psychiatric screening procedures between 2022 and 2023–2024, except for the analgesic intervention under evaluation. The absence of specific disclosure regarding the drug combination and PPD evaluation intent, and the post-hoc transition from quality improvement to research, represent limitations of this study.

### 2.2 Study population

We retrospectively reviewed electronic medical records to identify all parturients who underwent cesarean delivery and received the complete standardized multimodal analgesic protocol (both intraoperative esketamine 0.25 mg/kg and postoperative 48-h dexmedetomidine-esketamine-based PCIA) between January 2023 and December 2024. All 156 patients who received this protocol were treated uniformly with both components. From this population, we applied the following inclusion criteria to select the analytical cohort: maternal age 18–40 years; singleton pregnancy at ≥37 weeks gestation; American Society of Anesthesiologists (ASA) physical status II or III; and presence of at least one predefined high-risk factor for PPD, including a preoperative Edinburgh Postnatal Depression Scale (EPDS) score ≥9, documented history of depression or anxiety disorders, or exposure to major negative life events during pregnancy (e.g., spousal death, divorce, severe family conflict, or significant financial difficulties). Participants with two or more of these factors were categorized as having multiple high-risk factors.

Exclusion criteria were as follows: severe pregnancy-related complications (eclampsia, HELLP syndrome, or severe preeclampsia); significant hepatic, renal, or cardiovascular dysfunction; history of substance abuse; chronic use of antidepressant or anxiolytic medications within 1 month prior to surgery; postoperative intensive care unit admission or requirement for reoperation; neonatal severe malformations or birth asphyxia (Apgar score <7 at 5 min); or incomplete medical records or loss to follow-up at 6 weeks postpartum.

### 2.3 Anesthetic and analgesic protocol

All participants received standardized combined spinal-epidural anesthesia or general anesthesia when clinically indicated. Following fetal delivery and umbilical cord clamping, the standardized intervention was initiated. The control regimen (sufentanil-only PCIA) represented institutional standard care during 2022. The intervention regimen was designed as a complete protocol replacement rather than an incremental add-on study, reflecting real-world quality improvement implementation where changing entire analgesic paradigms is more feasible than testing individual components sequentially. During the intraoperative phase, esketamine 0.25 mg/kg was diluted in normal saline to a total volume of 20 mL and administered intravenously over 20 min. Postoperative analgesia commenced immediately upon arrival at the post-anesthesia care unit using patient-controlled intravenous analgesia (PCIA). The solution contained dexmedetomidine 2 μg/kg, esketamine 0.5 mg/kg, sufentanil 2 μg/kg, and tropisetron 10 mg diluted in normal saline to 100 mL. The pump was programmed to deliver a background infusion of 2 mL/h, with a bolus dose of 2 mL and a lockout interval of 15 min. The PCIA device was used for 48 h postoperatively. Rescue analgesia with flurbiprofen axetil 50 mg intravenously was provided upon request when the numerical rating scale (NRS) pain score was ≥4. Tropisetron was included in the intervention protocol primarily to prevent nausea/vomiting associated with esketamine use. We acknowledge this creates an asymmetry with the control group; however, excluding tropisetron would have compromised intervention tolerability and adherence, potentially introducing selection bias against the multimodal protocol.

### 2.4 Data collection and outcome measures

Data were retrospectively extracted from electronic medical records, anesthesia information systems, and postoperative follow-up databases by two independent researchers using a standardized data collection form. Discrepancies were resolved by consensus or consultation with a third reviewer.

EPDS and PSQI scores were prospectively collected through a routine clinical follow-up protocol (implemented January 2021) for all cesarean delivery patients: baseline EPDS preoperatively; postoperative EPDS at weeks 4 and 6; PSQI at day 7. Trained nursing staff administered these assessments via telephone or clinic visits and documented scores in the EMR. The 2022 historical controls underwent identical procedures. These routinely collected data were retrospectively extracted for analysis.

The primary outcome was the change in EPDS score from baseline to 6 weeks postpartum and the incidence of PPD at 6 weeks, defined as an EPDS score >10. The EPDS is a validated 10-item self-reported questionnaire for screening antenatal and postpartum depression, with scores ranging from 0 to 30 (higher scores indicate more severe depressive symptoms). EPDS scores were additionally assessed at postoperative week 4 as an interim time point to characterize the trajectory of depressive symptom changes.

Secondary outcomes included: (1) sleep quality at postoperative day 7, assessed by the Pittsburgh Sleep Quality Index (PSQI), a 19-item questionnaire with global scores from 0 to 21 (higher scores indicate poorer sleep quality); (2) pain intensity at rest and during movement at 6, 24, and 48 h postoperatively, measured using an 11-point NRS (0 = no pain, 10 = worst imaginable pain); (3) cumulative PCIA consumption and number of patient demands/valid deliveries; (4) exploratory biomarker assessment (IL-6, TNF-α, BDNF) in a convenience sample of patients with available residual serum samples; and (5) adverse events, including nausea, vomiting, dizziness, nightmares, hallucinations, sedation (Ramsay Sedation Scale ≥4), hypotension (systolic blood pressure <90 mmHg or >20% decrease from baseline), bradycardia (heart rate <50 beats/min), and respiratory depression (respiratory rate <10 breaths/min or oxygen saturation <93%).

### 2.5 Exploratory outcomes

The following recovery-related outcomes were retrospectively extracted from medical records to comprehensively evaluate the intervention’s impact: (1) Duration of hospital stay; (2) Time to first flatus and ambulation; (3) Breastfeeding initiation (≤24 h) and exclusive breastfeeding at 6 weeks; (4) Maternal satisfaction score (0–10 scale).

Baseline demographic and clinical variables included maternal age, body mass index (BMI), gestational age, parity, educational level, marital status, socioeconomic status, type of surgery (elective vs. emergency), duration of surgery, estimated blood loss, and neonatal birth weight.

### 2.6 Historical control group

To contextualize our findings, we utilized a historical control group comprising high-risk parturients who met identical high-risk criteria and underwent cesarean delivery at our institution during 2022, prior to implementation of the current multimodal analgesic protocol. These patients received standard-of-care PCIA with sufentanil alone (without dexmedetomidine or esketamine). Data were extracted using identical methodology and sources.

### 2.7 Sample size and statistical analysis

Based on institutional data indicating approximately 150 parturients undergoing cesarean delivery would receive the multimodal analgesic protocol during the 24-month enrollment period, and assuming 70% would meet the predefined high-risk research criteria and have complete follow-up data, we anticipated a final sample size of 80–100 participants. This provides 80% power to detect a standardized mean difference of 0.5 in EPDS score change (moderate effect size) at a two-sided significance level of 0.05.

We prospectively designated a single primary outcome (PPD incidence at 6 weeks) for confirmatory hypothesis testing. All secondary outcomes—including pain scores at multiple time points, sleep quality, biomarkers, analgesic consumption, recovery parameters, and adverse events—were designated as exploratory. No formal correction for multiple comparisons was applied to secondary outcomes; these findings are interpreted as hypothesis-generating, requiring confirmation in prospective trials with pre-specified hierarchical testing.

Continuous variables were tested for normality using the Shapiro–Wilk test. Normally distributed data are presented as mean ± standard deviation (SD) and were analyzed using paired *t*-tests (within-group) or independent *t*-tests (vs. historical controls). Non-normally distributed data are presented as median [interquartile range (IQR)] and were analyzed using Wilcoxon signed-rank tests (paired) or Mann–Whitney *U* tests (independent). Categorical variables are presented as frequency (percentage) and were compared using chi-square or Fisher’s exact tests. Effect sizes were calculated as Cohen’s *d* (continuous) and risk ratios (RR) with 95% confidence intervals (CI) (categorical). Multivariable logistic regression was performed to identify independent predictors of PPD at 6 weeks postpartum. Variables with *p* < 0.10 in univariate analysis were entered into the model using backward stepwise elimination. The final model included preoperative EPDS score, parity, mode of delivery (elective vs. emergency), estimated blood loss, cumulative PCIA consumption, and postoperative IL-6 levels. Results are reported as adjusted odds ratios (aOR) with 95% CI. Missing data were handled using multiple imputation by chained equations (MICE) for variables with <20% missingness, assuming missing at random. Sensitivity analyses were performed to assess the robustness of findings: (1) excluding participants with pre-existing psychiatric diagnoses; (2) using alternative EPDS cutoffs (≥12 vs. >10) for PPD definition; (3) complete case analysis; (4) alternative missing data approaches; and (5) per-protocol analysis excluding participants with protocol deviations. Analyses were performed using R version 4.3.0. A two-sided *p*-value <0.05 was considered statistically significant.

## 3 Results

### 3.1 Participant flow and baseline characteristics

**Figure 1:**
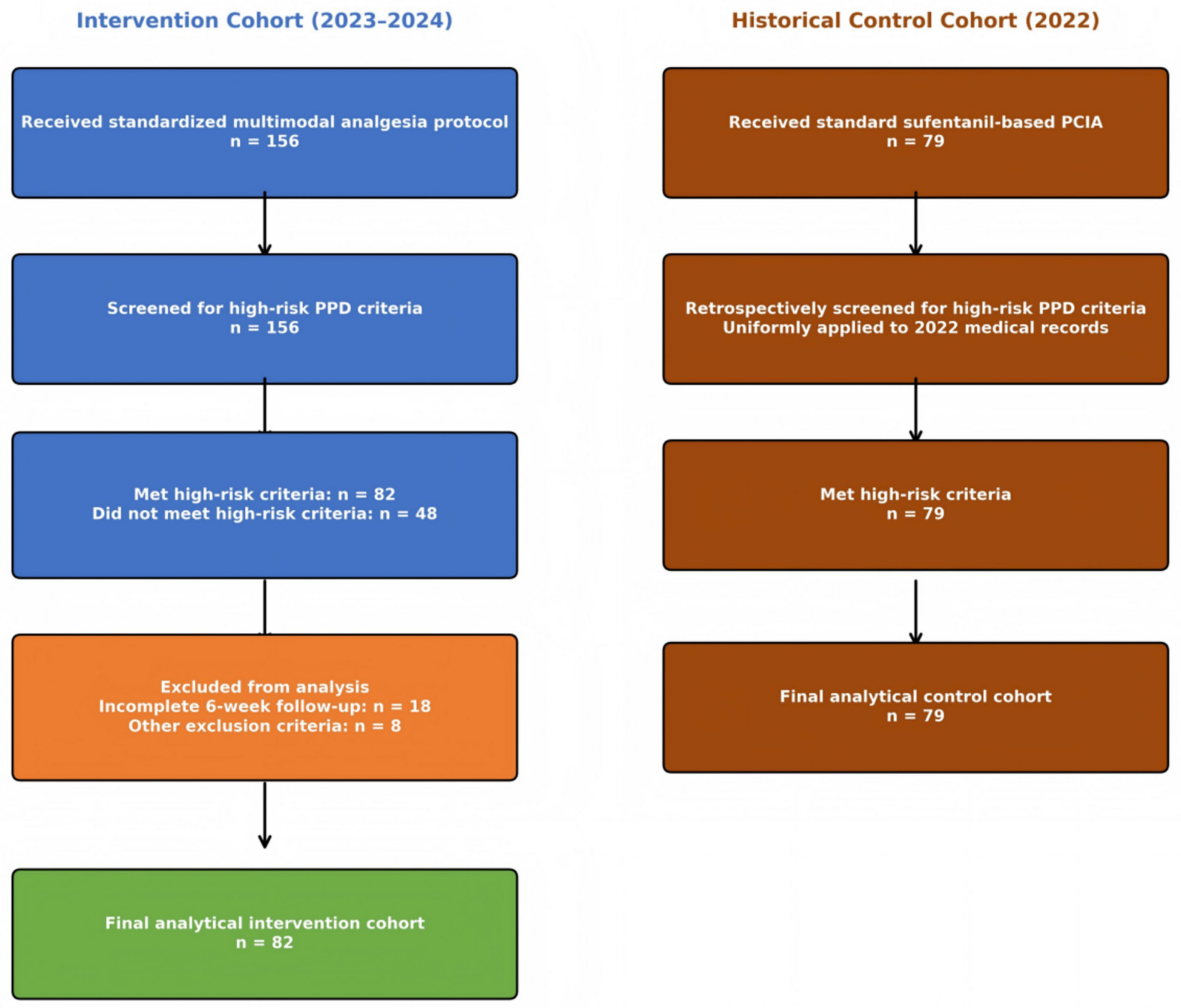
STROBE participant flow diagram of the study population.

Between January 2023 and December 2024, a total of 156 parturients undergoing cesarean delivery received the standardized multimodal analgesic protocol. Of these, 82 (52.6%) met the predefined high-risk research criteria and had complete 6-week follow-up data available for analysis. The remaining 74 were excluded due to: not meeting specific high-risk research criteria (*n* = 48, 30.8%)—these parturients received the intervention based on broader clinical indications but lacked the three specific research-defined risk factors; incomplete follow-up data at 6 weeks postpartum (*n* = 18); or other exclusion criteria (*n* = 8). Excluded patients (*n* = 48) were younger (29.2 ± 3.8 vs. 31.4 ± 4.2 years, *p* = 0.008) with lower baseline EPDS scores [6 (4–8) vs. 11 (9–13), *p* < 0.001] than included patients. Among 45 excluded patients with available follow-up data, PPD incidence was 8.9% (4/45) versus 14.6% (12/82) in the high-risk cohort, consistent with their lower-risk profile (Figure 1).

The historical control group comprised 79 high-risk parturients who underwent cesarean delivery at our institution during 2022 and received standard-of-care sufentanil-based patient-controlled intravenous analgesia without dexmedetomidine or esketamine.

Baseline demographic and clinical characteristics were comparable between the study cohort and historical controls (Table 1). The mean maternal age was 31.4 ± 4.2 years in the study cohort versus 30.8 ± 4.6 years in the control group (*p* = 0.38). Body mass index, gestational age, parity distribution, educational level, and socioeconomic status did not differ significantly between groups. The prevalence of specific high-risk factors for postpartum depression was similar: preoperative Edinburgh Postnatal Depression Scale score ≥9 (67.1% vs. 63.3%, *p* = 0.62), documented history of depression or anxiety disorders (31.7% vs. 29.1%, *p* = 0.73), and exposure to major negative life events during pregnancy (25.6% vs. 27.8%, *p* = 0.75). The proportion of emergency cesarean deliveries was slightly higher in the study cohort compared to controls (41.5% vs. 32.9%), though this difference did not reach statistical significance (*p* = 0.25).

**Table 1:** Baseline demographic and clinical characteristics.

Characteristic	Study cohort (*n* = 82)	Historical controls (*n* = 79)	*p*-value
Demographics			
Maternal age, years	31.4 ± 4.2	30.8 ± 4.6	0.38
Body mass index, kg/m^2^	28.6 ± 3.4	28.1 ± 3.8	0.41
Gestational age, weeks	38.7 ± 1.2	38.5 ± 1.3	0.29
Nulliparous, *n* (%)	45 (54.9)	41 (51.9)	0.71
Socioeconomic factors			
College education or higher, *n* (%)	38 (46.3)	35 (44.3)	0.81
Low socioeconomic status, *n* (%)	22 (26.8)	24 (30.4)	0.62
High-risk factors for PPD			
Preoperative EPDS score ≥9, *n* (%)	55 (67.1)	50 (63.3)	0.62
History of depression/anxiety, *n* (%)	26 (31.7)	23 (29.1)	0.73
Major negative life events, *n* (%)	21 (25.6)	22 (27.8)	0.75
Multiple high-risk factors, *n* (%)	18 (22.0)	16 (20.3)	0.79
Obstetric and surgical factors			
Emergency cesarean delivery, *n* (%)	34 (41.5)	26 (32.9)	0.25
Duration of surgery, min	52.3 ± 15.6	49.8 ± 14.2	0.28
Estimated blood loss, mL	285 ± 112	298 ± 128	0.49
Neonatal birth weight, g	3,245 ± 428	3,198 ± 415	0.51

Data are presented as mean ± standard deviation or number (percentage). EPDS, Edinburgh Postnatal Depression Scale; PPD, postpartum depression. *p*-values were calculated using independent *t*-tests for continuous variables and chi-square or Fisher’s exact tests for categorical variables.

### 3.2 Reduced postpartum depression incidence with multimodal analgesia

**Figure 2:**
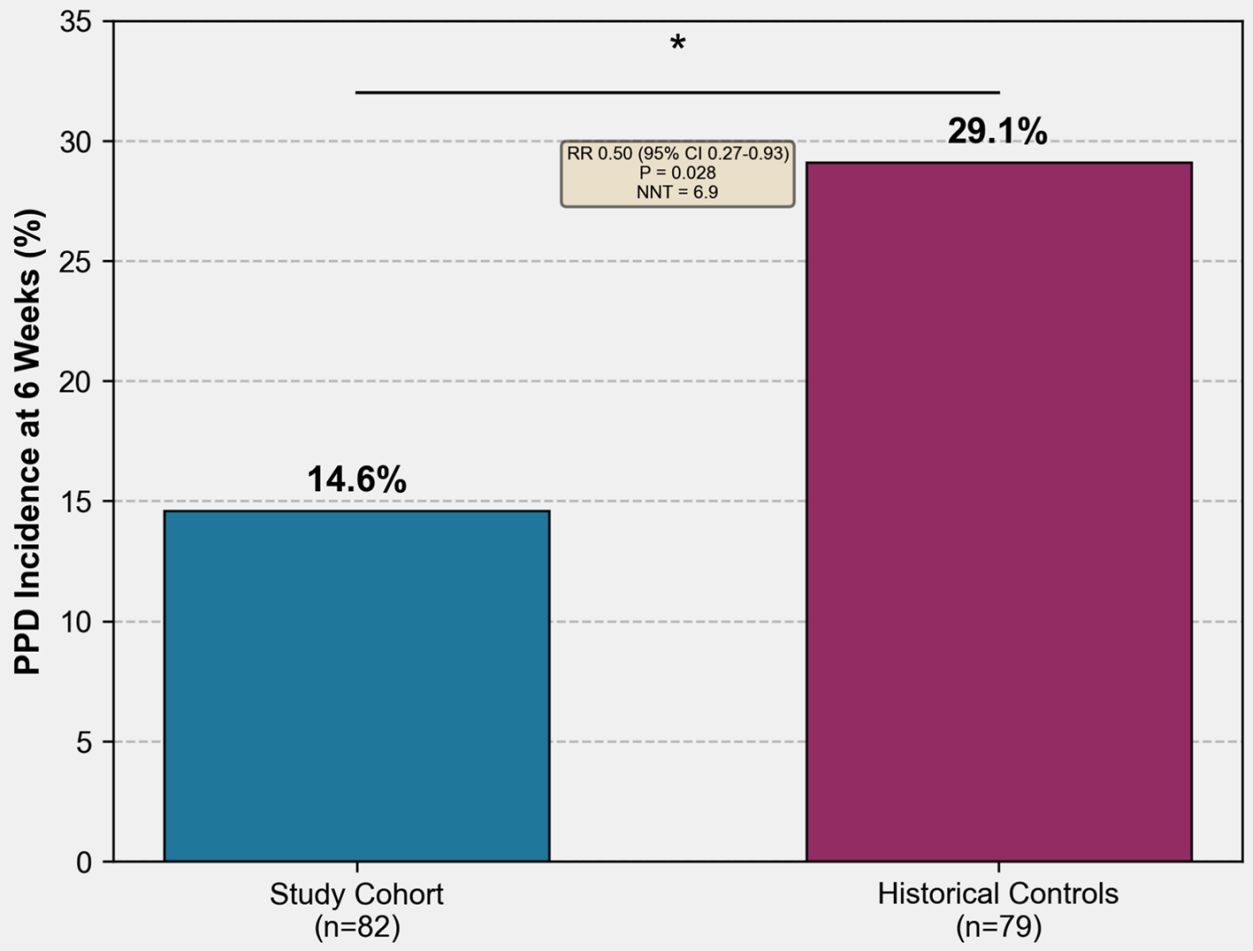
Incidence of postpartum depression at 6 weeks postpartum. Bar graph showing PPD incidence: study cohort 14.6% vs. historical controls 29.1%, with relative risk 0.50 (95% CI 0.27–0.93), *p* = 0.028, and NNT = 6.9.

The primary outcome analysis revealed a significantly lower incidence of postpartum depression at 6 weeks postpartum in the study cohort compared to historical controls. Postpartum depression, defined as an Edinburgh Postnatal Depression Scale score >10, was observed in 12 of 82 participants (14.6%) in the study cohort versus 23 of 79 participants (29.1%) in the control group, representing a relative risk reduction of 49.8% (relative risk 0.50, 95% confidence interval 0.27 to 0.93, *p* = 0.028, number needed to treat 6.9) (Figure 2).

The mean change in Edinburgh Postnatal Depression Scale score from baseline to 6 weeks postpartum demonstrated a significant improvement in the study cohort compared to controls. Participants in the study cohort exhibited a mean decrease in Edinburgh Postnatal Depression Scale score of 2.8 ± 3.4 points, whereas controls showed a mean increase of 0.6 ± 4.1 points, yielding a between-group difference of 3.4 points (95% confidence interval 1.9 to 4.9, *p* < 0.001, Cohen’s *d* = 0.91) (Table 2 and Supplementary Figure 1). Within-group analysis confirmed that the reduction in Edinburgh Postnatal Depression Scale score in the study cohort was statistically significant (paired *t*-test, *p* < 0.001), whereas the modest increase observed in controls did not reach significance (*p* = 0.19).

**Table 2:** Primary and key secondary outcomes.

Outcome	Study cohort (*n* = 82)	Historical controls (*n* = 79)	Between-group difference (95% CI)	*p*-value	Effect size
Primary outcome					
PPD at 6 weeks, *n* (%)	12 (14.6)	23 (29.1)	RR 0.50 (0.27–0.93)	0.028	—
Change in EPDS score (6 weeks—baseline)	−2.8 ± 3.4	+0.6 ± 4.1	3.4 (1.9–4.9)	<0.001	Cohen’s *d* = 0.91
Key secondary outcomes					
PSQI score at day 7	6.2 ± 2.8	8.9 ± 3.1	2.7 (1.6–3.8)	<0.001	Cohen’s *d* = 0.92
Poor sleep quality (PSQI >5), *n* (%)	40 (48.8)	61 (77.2)	RR 0.63 (0.48–0.83)	0.001	—
IL-6 at 24 h, pg/mL	28.4 (19.2–42.6)	45.7 (32.1–68.3)	—	<0.001	*r* = 0.42
BDNF at 24 h, pg/mL	8,420 (6,180–11,340)	5,640 (4,020–7,890)	—	<0.001	*r* = 0.38

Data are presented as mean ± standard deviation, median (interquartile range), or number (percentage). EPDS, Edinburgh Postnatal Depression Scale; PPD, postpartum depression defined as EPDS >10; PSQI, Pittsburgh Sleep Quality Index; IL-6, interleukin-6; BDNF, brain-derived neurotrophic factor; RR, relative risk; CI, confidence interval. Effect sizes: Cohen’s *d* for continuous variables, Spearman’s *r* for non-parametric comparisons. Biomarker data available in convenience sample only (*n* = *X* intervention, *n* = *Y* controls); presented as exploratory.

### 3.3 Secondary outcomes: multidimensional benefits of the intervention

#### 3.3.1 Improved early postoperative sleep quality

Sleep quality at postoperative day 7, assessed using the Pittsburgh Sleep Quality Index, was significantly better in the study cohort compared to historical controls. The mean global Pittsburgh Sleep Quality Index score was 6.2 ± 2.8 in the study cohort versus 8.9 ± 3.1 in controls (between-group difference 2.7 points, 95% confidence interval 1.6 to 3.8, *p* < 0.001, Cohen’s *d* = 0.92). The proportion of participants with poor sleep quality, defined as Pittsburgh Sleep Quality Index score >5, was 48.8% in the study cohort compared to 77.2% in controls (relative risk 0.63, 95% confidence interval 0.48 to 0.83, *p* = 0.001) (Table 2 and Supplementary Figure 2).

#### 3.3.2 Enhanced postoperative analgesia with reduced opioid consumption

Pain intensity scores at rest and during movement were consistently lower in the study cohort at all assessed time points (Table 3 and Supplementary Figure 3). At 6 h postoperatively, the mean numerical rating scale score at rest was 2.1 ± 0.9 in the study cohort versus 3.4 ± 1.2 in controls (*p* < 0.001), and during movement was 3.8 ± 1.3 versus 5.2 ± 1.6 (*p* < 0.001). These differences persisted at 24 and 48 h postoperatively, with effect sizes ranging from 0.82 to 1.21.

**Table 3:** Postoperative pain scores and analgesic consumption.

Variable	Study cohort (*n* = 82)	Historical controls (*n* = 79)	*p*-value	Effect size (Cohen’s *d*)
NRS pain score at rest				
6 h	2.1 ± 0.9	3.4 ± 1.2	<0.001	1.21
24 hours	1.8 ± 0.8	2.9 ± 1.0	<0.001	1.22
48 hours	1.5 ± 0.7	2.3 ± 0.9	<0.001	0.99
NRS pain score with movement				
6 h	3.8 ± 1.3	5.2 ± 1.6	<0.001	0.96
24 h	3.2 ± 1.1	4.5 ± 1.4	<0.001	1.03
48 h	2.6 ± 1.0	3.4 ± 1.2	<0.001	0.82
PCIA consumption (48 h)				
Cumulative volume, mL	156 (134–178)	198 (172–231)	<0.001	*r* = 0.45
Patient demands, *n*	8 (5–12)	14 (10–19)	<0.001	*r* = 0.48
Valid deliveries, *n*	6 (4–9)	11 (7–15)	<0.001	*r* = 0.46

Data are presented as mean ± standard deviation or median (interquartile range). NRS, Numerical Rating Scale (0–10); PCIA, patient-controlled intravenous analgesia.

Cumulative patient-controlled intravenous analgesia consumption during the 48-h postoperative period was significantly reduced in the study cohort. The median cumulative volume infused was 156 mL (interquartile range 134 to 178 mL) in the study cohort compared to 198 mL (interquartile range 172 to 231 mL) in controls (*p* < 0.001), representing a 21.2% relative reduction in median consumption. The median number of patient demands was 8 (interquartile range 5 to 12) versus 14 (interquartile range 10 to 19, *p* < 0.001), and the median number of valid deliveries was 6 (interquartile range 4 to 9) versus 11 (interquartile range 7 to 15, *p* < 0.001) (Supplementary Figure 4). The 21.2% reduction in PCIA consumption occurred in the context of identical sufentanil concentration (2 μg/kg per 100 mL) and pump programming across groups. Whether this opioid-sparing effect contributes to observed benefits through reduced opioid-related adverse effects, or represents a confound of differential pharmacological exposure, cannot be determined in this design.

#### 3.3.3 Exploratory biomarker observations

**Figure 3:**
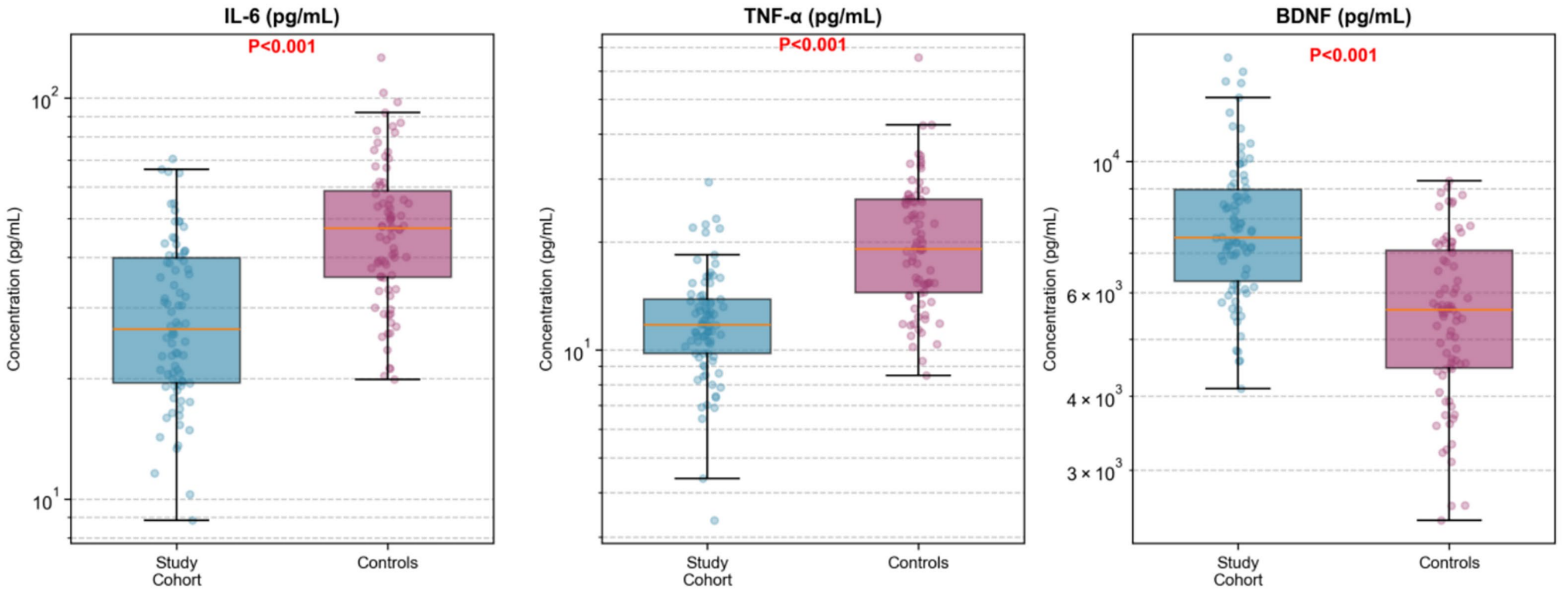
Serum biomarker profiles at 24 hours postoperatively. Three-panel box plot or dot plot showing distributions of IL-6, TNF-α, and BDNF. Study cohort shows lower IL-6 and TNF-α, higher BDNF compared to controls. Individual data points overlaid.

In a subset of patients with available residual serum samples, exploratory biomarker analysis suggested favorable modulation in the study cohort compared with controls (Table 2 and Figure 3). Interleukin-6 levels were lower in the study cohort (median 28.4 pg/mL, IQR 19.2–42.6) compared to controls (median 45.7 pg/mL, IQR 32.1–68.3, *p* < 0.001). Similarly, tumor necrosis factor-alpha levels were reduced in the study cohort (median 12.8 pg/mL, IQR 8.4–19.2) versus controls (median 21.4 pg/mL, IQR 14.6–31.7, *p* < 0.001). Brain-derived neurotrophic factor levels were higher in the study cohort (median 8,420 pg/mL, IQR 6,180–11,340) compared to controls (median 5,640 pg/mL, IQR 4,020–7,890, *p* < 0.001). These observations are exploratory due to non-systematic sample availability and should not be interpreted as confirmatory.

#### 3.3.4 Accelerated postoperative recovery and maternal-infant outcomes

The multimodal analgesic protocol was associated with enhanced postoperative recovery parameters (Table 4). The median duration of hospital stay was 3.0 days (interquartile range 2.0 to 4.0 days) in the study cohort versus 3.5 days (interquartile range 3.0 to 4.5 days) in controls (*p* = 0.042). Time to first flatus was shorter in the study cohort (median 18.5 h, interquartile range 14.0 to 24.0 h) compared to controls (median 24.0 h, interquartile range 19.5 to 30.0 h, *p* = 0.003). Breastfeeding initiation within 24 h postoperatively was achieved in 89.0% of participants in the study cohort versus 78.5% in controls, though this difference did not reach statistical significance (*p* = 0.08).

**Table 4:** Postoperative recovery and maternal-infant outcomes.

Outcome	Study cohort (*n* = 82)	Historical controls (*n* = 79)	*p*-value
Hospital stay, days	3.0 (2.0–4.0)	3.5 (3.0–4.5)	0.042
Time to first flatus, hours	18.5 (14.0–24.0)	24.0 (19.5–30.0)	0.003
Time to ambulation, hours	14.2 ± 4.6	16.8 ± 5.2	0.001
Breastfeeding initiation ≤24 h, *n* (%)	73 (89.0)	62 (78.5)	0.08
Exclusive breastfeeding at 6 weeks, *n* (%)	58 (70.7)	48 (60.8)	0.16
Maternal satisfaction score (0–10)	8.4 ± 1.2	7.1 ± 1.5	<0.001

Data are presented as mean ± standard deviation, median (interquartile range), or number (percentage).

### 3.4 Safety profile: acceptable tolerability of the multimodal protocol

Adverse events were assessed in all 156 patients who received the multimodal protocol (Table 5). Psychotomimetic effects (nightmares and dissociative symptoms) occurred in 13.5% (21/156) of the full cohort and 8.5% (7/82) of the high-risk subgroup, all transient and self-limited. Nausea and vomiting were 23.1% (36/156) and 12.2% (19/156) in the full cohort, comparable to the high-risk subgroup and historical controls. Cardiovascular and respiratory safety profiles were acceptable, with hypotension in 7.1% (11/156), bradycardia in 3.8% (6/156), and no respiratory depression. Excessive sedation occurred in 5.1% (8/156). No significant differences were observed between the full treated cohort and high-risk subgroup.

**Table 5:** Adverse events and safety outcomes.

Adverse event	Full cohort (*n* = 156)	High-risk cohort (*n* = 82)	Historical controls (*n* = 79)
Gastrointestinal			
Nausea, *n* (%)	36 (23.1)	19 (23.2)	21 (26.6)
Vomiting, *n* (%)	19 (12.2)	10 (12.2)	12 (15.2)
Neurological/Psychological			
Dizziness, *n* (%)	29 (18.6)	15 (18.3)	9 (11.4)
Headache, *n* (%)	15 (9.6)	8 (9.8)	7 (8.9)
Nightmares, *n* (%)	12 (7.7)	4 (4.9)	2 (2.5)
Dissociative symptoms, *n* (%)	9 (5.8)	3 (3.7)	0 (0)
Hallucinations, *n* (%)	0 (0)	0 (0)	0 (0)
Cardiovascular			
Hypotension, *n* (%)	11 (7.1)	6 (7.3)	7 (8.9)
Bradycardia, *n* (%)	6 (3.8)	3 (3.7)	4 (5.1)
Respiratory			
Respiratory depression, *n* (%)	0 (0)	0 (0)	0 (0)
Oxygen desaturation (<93%), *n* (%)	4 (2.6)	2 (2.4)	3 (3.8)
Sedation			
Excessive sedation (RSS ≥4), *n* (%)	8 (5.1)	4 (4.9)	2 (2.5)
Severe sedation (RSS = 6), *n* (%)	0 (0)	0 (0)	0 (0)

RSS, Ramsay Sedation Scale.

### 3.5 Association between postpartum depression and treatment effect modification

Multivariable logistic regression analysis was performed to identify independent predictors of postpartum depression at 6 weeks postpartum in the combined cohort (Table 6). After adjustment for potential confounders, receipt of the multimodal analgesic protocol remained significantly associated with reduced odds of postpartum depression (adjusted odds ratio 0.38, 95% confidence interval 0.16 to 0.89, *p* = 0.026). Additional independent predictors included preoperative Edinburgh Postnatal Depression Scale score (adjusted odds ratio 1.18 per point increase, 95% confidence interval 1.08 to 1.29, *p* < 0.001), emergency cesarean delivery (adjusted odds ratio 2.42, 95% confidence interval 1.12 to 5.24, *p* = 0.025), and postoperative interleukin-6 level (adjusted odds ratio 1.03 per 10 pg/mL increase, 95% confidence interval 1.01 to 1.05, *p* = 0.008). Parity, estimated blood loss, and cumulative patient-controlled intravenous analgesia consumption were not independently associated with postpartum depression in the final model.

**Table 6:** Multivariable logistic regression analysis of predictors for postpartum depression at 6 weeks.

Variable	Adjusted OR (95% CI)	*p*-value
Multimodal analgesic protocol (vs. standard care)	0.38 (0.16–0.89)	0.026
Preoperative EPDS score (per 1-point increase)	1.18 (1.08–1.29)	<0.001
Emergency cesarean delivery (vs. elective)	2.42 (1.12–5.24)	0.025
Age ≥35 years (vs. <35 years)	1.56 (0.72–3.38)	0.26
Nulliparous (vs. multiparous)	1.28 (0.61–2.69)	0.51
History of depression/anxiety	1.45 (0.68–3.12)	0.34
Estimated blood loss (per 100 mL increase)	1.02 (0.98–1.06)	0.38
Cumulative PCIA consumption (per 10 mL increase)	1.01 (0.99–1.03)	0.42

OR, odds ratio; CI, confidence interval; EPDS, Edinburgh Postnatal Depression Scale; IL-6, interleukin-6; PCIA, patient-controlled intravenous analgesia. The model was adjusted for all variables listed in the table. Hosmer–Lemeshow test *p* = 0.72, indicating adequate model fit.

Subgroup analyses were conducted to evaluate the consistency of treatment effects across predefined subgroups (Supplementary Figure 5). The protective effect of the multimodal analgesic protocol against postpartum depression was consistent across subgroups defined by age (<35 vs. ≥35 years), parity (nulliparous vs. multiparous), mode of delivery (elective vs. emergency), type of high-risk factor (preoperative Edinburgh Postnatal Depression Scale score vs. psychiatric history vs. negative life events), and baseline Edinburgh Postnatal Depression Scale score (<12 vs. ≥12). No significant interaction effects were observed (all *p* for interaction >0.10).

### 3.6 Robustness of findings across alternative analytical approaches

Sensitivity analyses yielded consistent results with the primary analysis (Table 7). When participants with pre-existing psychiatric diagnoses were excluded, the incidence of postpartum depression was 11.4% (7 of 61) in the study cohort versus 27.5% (16 of 58) in controls (relative risk 0.41, 95% confidence interval 0.19 to 0.91, *p* = 0.025). Using an alternative Edinburgh Postnatal Depression Scale cutoff of ≥12 to define postpartum depression, the incidence was 9.8% (8 of 82) in the study cohort versus 22.8% (18 of 79) in controls (relative risk 0.43, 95% confidence interval 0.20 to 0.93, *p* = 0.031). Analysis of complete cases without multiple imputation (*n* = 78 study cohort, *n* = 75 controls) confirmed the primary findings (relative risk 0.48, 95% confidence interval 0.25 to 0.91, *p* = 0.024).

**Table 7:** Sensitivity analyses for primary outcome.

Analysis	Study cohort	Historical controls	RR (95% CI)	*p*-value
Primary analysis	12/82 (14.6%)	23/79 (29.1%)	0.50 (0.27–0.93)	0.028
Excluding psychiatric history	7/61 (11.4%)	16/58 (27.5%)	0.41 (0.19–0.91)	0.025
EPDS cutoff ≥12 for PPD	8/82 (9.8%)	18/79 (22.8%)	0.43 (0.20–0.93)	0.031
Complete case analysis	11/78 (14.1%)	22/75 (29.3%)	0.48 (0.25–0.91)	0.024
Multiple imputation (*m* = 20)	12.3/82 (15.0%)	23.1/79 (29.2%)	0.51 (0.28–0.94)	0.030
Per-protocol analysis	11/79 (13.9%)	—	—	—

RR, relative risk; CI, confidence interval; EPDS, Edinburgh Postnatal Depression Scale; PPD, postpartum depression.

### 3.7 Exploratory correlation analyses

**Figure 4:**
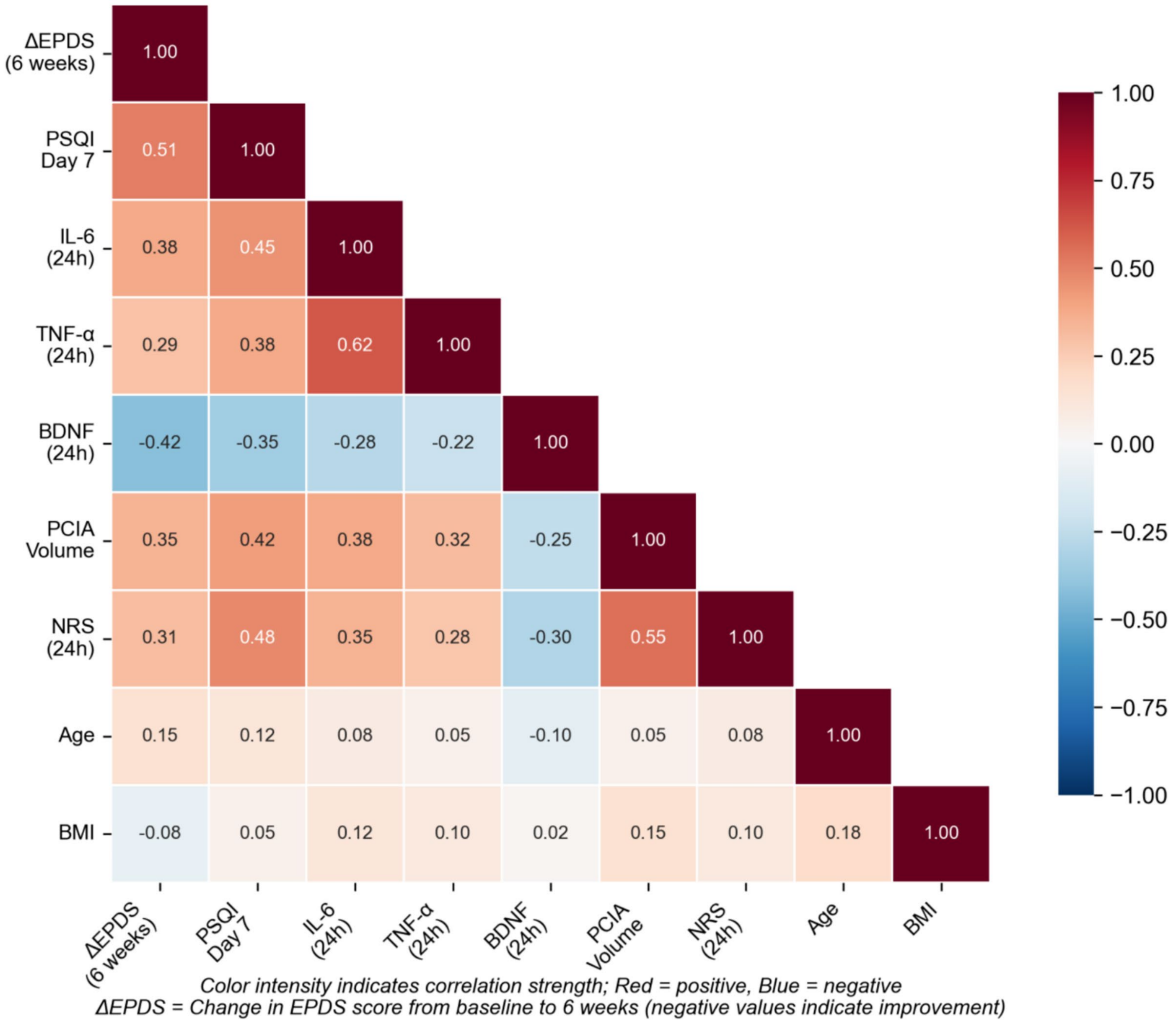
Correlation matrix of key biological and clinical variables. Heatmap or correlogram showing Spearman correlation coefficients between variables: ΔEPDS, PSQI, IL-6, TNF-α, BDNF, PCIA consumption, pain scores. Color-coded to show direction and strength of correlations. Exploratory: biomarker data available in subset only.

Correlation analyses were performed to explore relationships between key variables in the study cohort (Figure 4 and Supplementary Table 1). The change in EPDS score demonstrated moderate negative correlation with PSQI score at day 7 (Spearman’s rho = 0.51, *p* < 0.001) and moderate positive correlation with cumulative PCIA consumption (Spearman’s rho = 0.35, *p* = 0.001). Exploratory correlations with biomarkers (available in subset only) suggested associations between improved depressive symptoms and lower IL-6 (rho = 0.38) and higher BDNF (rho = −0.42), though these should be interpreted cautiously given limited sample availability.

## 4 Discussion

This retrospective cohort study provides preliminary evidence that a multimodal analgesic protocol combining intraoperative esketamine with postoperative dexmedetomidine-esketamine-based patient-controlled intravenous analgesia is associated with reduced postpartum depression incidence and improved early sleep quality in high-risk parturients undergoing cesarean delivery. The observed 49.8% relative risk reduction in PPD at 6 weeks, coupled with significant improvements in postoperative sleep architecture and attenuated inflammatory responses, suggests that this pharmacological strategy may intersect with multiple interconnected pathways implicated in PPD pathogenesis.

The primary finding of a 14.6% PPD incidence in the study cohort compared with 29.1% in historical controls aligns closely with the 10.4% reported in the recent randomized trial of intraoperative esketamine alone (12). This consistency is noteworthy given the differences in study design and suggests that esketamine’s antidepressant effects are robust across diverse clinical settings. However, the addition of dexmedetomidine was associated with incremental improvements in sleep optimization, as evidenced by the 2.7-point improvement in Pittsburgh Sleep Quality Index scores and the 28.4% absolute reduction in poor sleep quality prevalence. Given that sleep disturbance is both a risk factor for and early manifestation of PPD (18, 19), the observed sleep improvements co-occurred with sustained effects, though causal direction cannot be established observed at 6 weeks. The analgesic properties of the protocol may contribute to observed benefits beyond specific antidepressant mechanisms. A recent editorial by Lucke-Wold and Karamian (20) highlights that inadequate post-cesarean pain management is associated with decreased maternal care, prolonged hospitalization, and elevated postpartum depression risk, suggesting that effective analgesia itself may modify PPD risk through multiple pathways. Esketamine’s potent analgesic effects, mediated through NMDA receptor antagonism and modulation of central sensitization, may modify the pain-depression association that characterizes the early postoperative period (20, 21). In our study, the 21.2% reduction in opioid consumption alongside superior pain scores suggests that the multimodal protocol’s analgesic efficacy may reduce opioid-related adverse effects while providing direct pain relief, both of which could be associated with improved postpartum outcomes. However, we acknowledge that our study design cannot disentangle analgesic effects from specific antidepressant or other pharmacological actions of the protocol.

The multimodal protocol produced a 21.2% reduction in PCIA utilization, consistent with known opioid-sparing effects of dexmedetomidine and esketamine. Whether observed benefits in postpartum depression derive from this reduced opioid exposure, from direct neuropharmacological actions of the adjunctive agents, or from improved control of opioid adverse effects via tropisetron cannot be disentangled. Esketamine’s rapid antidepressant effects are attributed to N-methyl-D-aspartate receptor antagonism, activation of mammalian target of rapamycin signaling, and enhanced BDNF release, which promote synaptic plasticity and reverse stress-induced neuronal atrophy (22, 23). Dexmedetomidine has been hypothesized to act through distinct yet synergistic pathways: its α2-adrenergic agonism produces analgesia without respiratory depression, reduces catecholamine-induced neuroinflammation, and uniquely promotes natural non-rapid eye movement sleep by activating endogenous sleep pathways in the ventrolateral preoptic nucleus (24, 25). The correlation analyses showed that improvements in depressive symptoms were associated with better sleep quality and reduced analgesic requirements. Exploratory biomarker observations, while limited by non-systematic sample availability, were directionally consistent with known anti-inflammatory and neurotrophic effects of dexmedetomidine and esketamine (26, 27). Sen et al. (28) previously established the critical link between serum BDNF levels, depression, and antidepressant treatment response in their meta-analysis, providing context for our biomarker findings.

We acknowledge that tropisetron’s antiemetic and potential anxiolytic effects may have contributed to observed outcomes. However, several considerations suggest this does not fully explain our findings: (1) the magnitude of PPD risk reduction (RR 0.50) exceeds effect sizes typically associated with antiemetic interventions alone; (2) biomarker changes (IL-6 reduction, BDNF elevation) are not consistent with pure 5-HT3 antagonism; (3) sleep quality improvements at day 7 are more consistent with dexmedetomidine’s pharmacological profile than with tropisetron. Nevertheless, we cannot quantify tropisetron’s specific contribution.

The substantial reduction in opioid consumption observed in our study carries important clinical implications beyond the primary psychiatric outcomes. Opioid-sparing analgesia reduces the risk of opioid-related adverse events, facilitates earlier ambulation, and may improve breastfeeding initiation (29, 30). The shorter time to first flatus and reduced hospital stay in the study cohort likely reflect the multimodal protocol’s favorable effects on gastrointestinal function and early recovery. These findings are consistent with enhanced recovery after surgery principles and suggest that the intervention may yield benefits across multiple domains of postoperative care.

Safety considerations are paramount when evaluating novel analgesic strategies in obstetric populations. The incidence of psychotomimetic adverse effects in our study (8.5%) was substantially lower than that reported with both anesthetic doses of ketamine (20–30%) and subanesthetic intranasal esketamine in antidepressant trials (27–61% for dissociation and sedation) (31, 32). All episodes were transient, self-limited, and did not require pharmacological intervention, suggesting acceptable tolerability for postpartum patients. The cardiovascular and respiratory safety profiles were reassuring, with no significant differences in hypotension, bradycardia, or respiratory depression compared with standard opioid-based analgesia. Notably, excessive sedation was infrequent and severe sedation was absent, supporting the feasibility of this protocol in general ward settings without intensive monitoring.

Our findings should be interpreted within the broader context of PPD risk factors and preventive strategies. The high-risk criteria employed in our study—preoperative EPDS score ≥9, documented history of depression/anxiety, and major negative life events—represent well-established risk factors for PPD. (33, 34) Shorey et al. (1) estimated global PPD prevalence at 10–20% among healthy mothers, with substantially higher rates in at-risk populations, consistent with the 29.1% incidence observed in our historical controls. The number needed to treat of 6.9 suggests that this intervention could have meaningful public health impact if implemented broadly.

The peripartum period represents a critical window for intervention, as emphasized by O’Hara and McCabe (5) in their comprehensive review of PPD pathophysiology and treatment. By targeting modifiable risk factors—acute pain, sleep disturbance, and neuroinflammation—during this vulnerable period, our multimodal approach aligns with contemporary preventive psychiatry principles (35).

Several limitations must be acknowledged. The protocol evolved from quality improvement to research without prospective trial registration or specific informed consent, constraining validity. The 2022 historical control period coincided with China’s Zero-COVID policy, which may have independently elevated depression rates through mechanisms overlapping with those targeted by the intervention—a fundamental temporal confound. While secular trends would predict lower PPD incidence in 2023–2024, observed benefit against this trend suggests robust signal, though definitive causal inference requires randomized trials. The retrospective, single-center design with historical controls precludes definitive causal inferences and introduces selection biases. The exclusion of 48 treated patients for not meeting high-risk criteria could introduce selection bias; however, these patients showed expected lower PPD rates (8.9% vs. 14.6%) given their lower baseline risk, arguing against selective exclusion of non-responders. The absence of randomization and blinding may have influenced treatment assignment, although standardized depression screening mitigates this concern. The intervention combined four active agents versus sufentanil alone, precluding isolation of individual drug effects and introducing confounding by: (a) tropisetron’s antiemetic/anxiolytic properties; (b) differential opioid exposure; and (c) pharmacodynamic interactions. Our findings demonstrate association of the protocol-as-a-whole with improved outcomes, not specific efficacy of individual components. The modest sample size (*n* = 82) limits statistical power for subgroup analyses and rare adverse event detection. The single-center design restricts generalizability. The 6-week follow-up does not capture longer-term trajectories or delayed adverse effects. Unmonitored neonatal drug exposure via breast milk during 48-h PCIA represents a patient safety gap; available lactation safety data are limited to single intraoperative doses, not continuous infusion. Exploratory biomarker analyses were limited to a non-systematic subset. We conducted approximately 25–30 statistical tests without formal correction for multiplicity; only PPD incidence was designated for confirmatory inference, with all secondary outcomes exploratory. This increases false-positive risk, particularly in a retrospective design. We have attempted to mitigate this by emphasizing consistency of directional effects across mechanistically related domains rather than reliance on isolated significant *p*-values.

Our findings in cesarean delivery patients may not directly translate to vaginal delivery populations, where pain mechanisms, inflammatory profiles, and postpartum recovery trajectories differ substantially. Whether multimodal analgesic strategies incorporating dexmedetomidine and esketamine would benefit high-risk parturients after vaginal delivery remains unknown and warrants prospective evaluation. Additionally, dosing optimization to achieve adequate analgesia while preserving maternal alertness for meaningful skin-to-skin contact and early breastfeeding represents a critical clinical challenge. The sedative properties of both dexmedetomidine and esketamine, while potentially beneficial for sleep quality, may interfere with maternal-infant bonding if not carefully titrated. Esketamine and dexmedetomidine may not be universally available. Simplified protocols using ketamine (where esketamine is inaccessible) or clonidine (as a partial dexmedetomidine alternative) warrant investigation. Non-pharmacological strategies (optimized breastfeeding support, early mobilization, cognitive-behavioral approaches) may offer incremental benefit when advanced agents are unavailable. Future research should evaluate stepped-care models that match intervention intensity to available resources, incorporate maternal-infant interaction outcomes and objective sedation monitoring, and employ dose-ranging designs to identify optimal regimens that balance analgesic efficacy with preserved maternal capacity for early postpartum care across diverse delivery populations.

## 5 Conclusion

A dexmedetomidine-esketamine multimodal analgesic protocol was associated with lower postpartum depression incidence, better sleep quality, and acceptable safety in high-risk parturients undergoing cesarean delivery. These findings support the hypothesis that with effects on pain, sleep, and inflammatory pathways through rational pharmacological combinations may represent a practical approach for reducing PPD risk. These findings are hypothesis-generating and require confirmation in prospective randomized trials with contemporaneous controls, particularly given the unresolvable temporal confounding introduced by the Zero-COVID policy transition.

## Data Availability

The original contributions presented in the study are included in the article/Supplementary material, further inquiries can be directed to the corresponding author.
