# Supplementary material for: Association of multimodal analgesic protocol with postpartum depression incidence and sleep quality in high-risk parturients
Source: Front Med (Lausanne). 2026 May 20;13:1828838. doi: 10.3389/fmed.2026.1828838 (PMC13229967; doi:10.3389/fmed.2026.1828838)
Supplement: Supplementary file 1 [file Supplementary_file_1.docx]

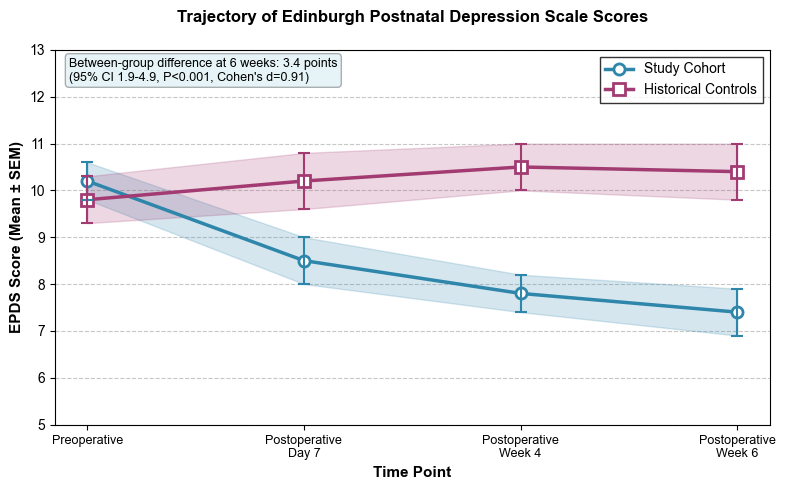


**Supplementary Figure 1**. Trajectory of Edinburgh Postnatal Depression Scale Scores. EPDS scores were assessed at four time points: preoperative, postoperative day 7, postoperative week 4 (exploratory interim assessment), and postoperative week 6. Week 4 was not prespecified in the study protocol but was added to characterize the time course of symptom changes between early and late postpartum periods. Study cohort shows decreasing trajectory from 10.2 to 7.4, while controls show slight increase from 9.8 to 10.4. Error bars represent standard deviation.


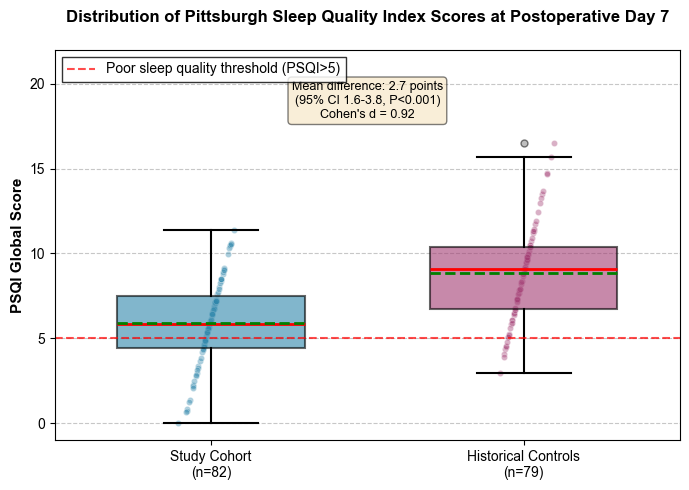


**Supplementary Figure 2**. Distribution of Pittsburgh Sleep Quality Index Scores at Postoperative Day 7

[Histogram or box plot showing distribution of PSQI scores, with median and interquartile range indicated. Study cohort shifted toward lower (better) scores compared to controls]


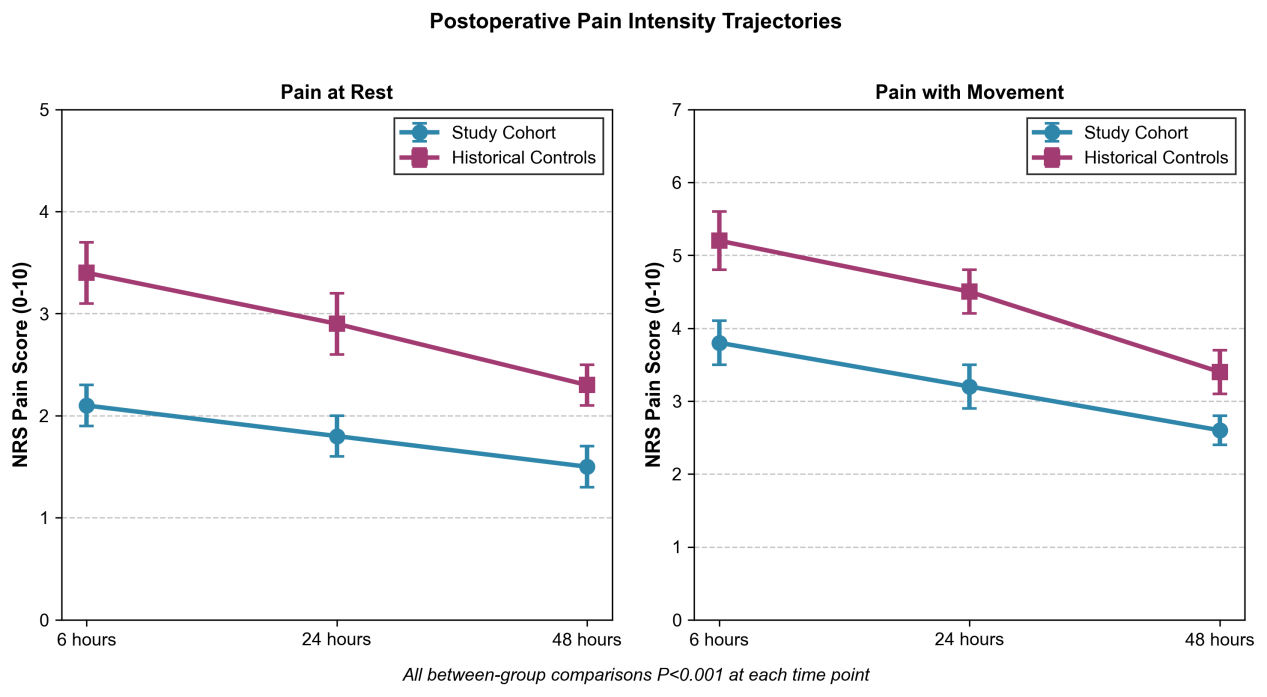


**Supplementary Figure 3**. Postoperative Pain Intensity Trajectories

[Line graph with four lines showing NRS scores at rest and with movement for both groups across three


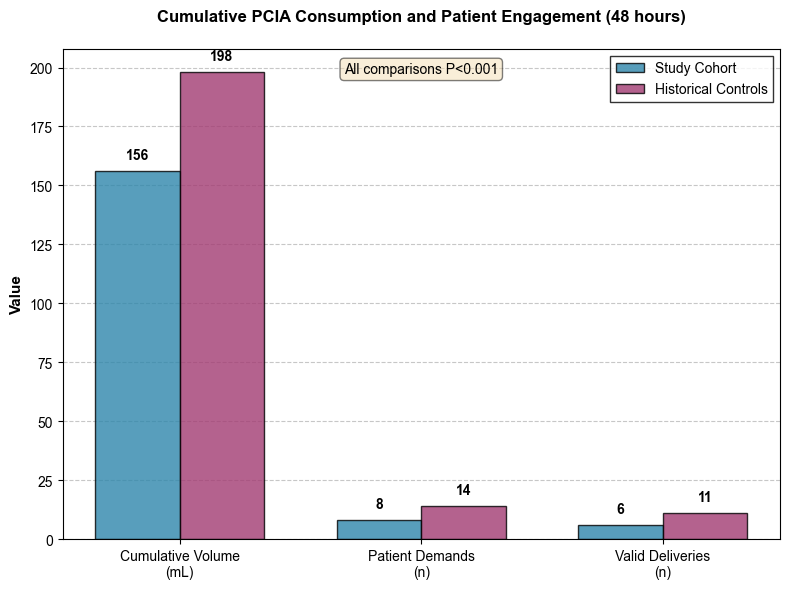


**Supplementary Figure 4.** Cumulative PCIA Consumption and Patient Engagement

[Bar graph or box plot showing three metrics: cumulative volume (mL), number of demands, and number of valid deliveries. All three significantly lower in study cohort]


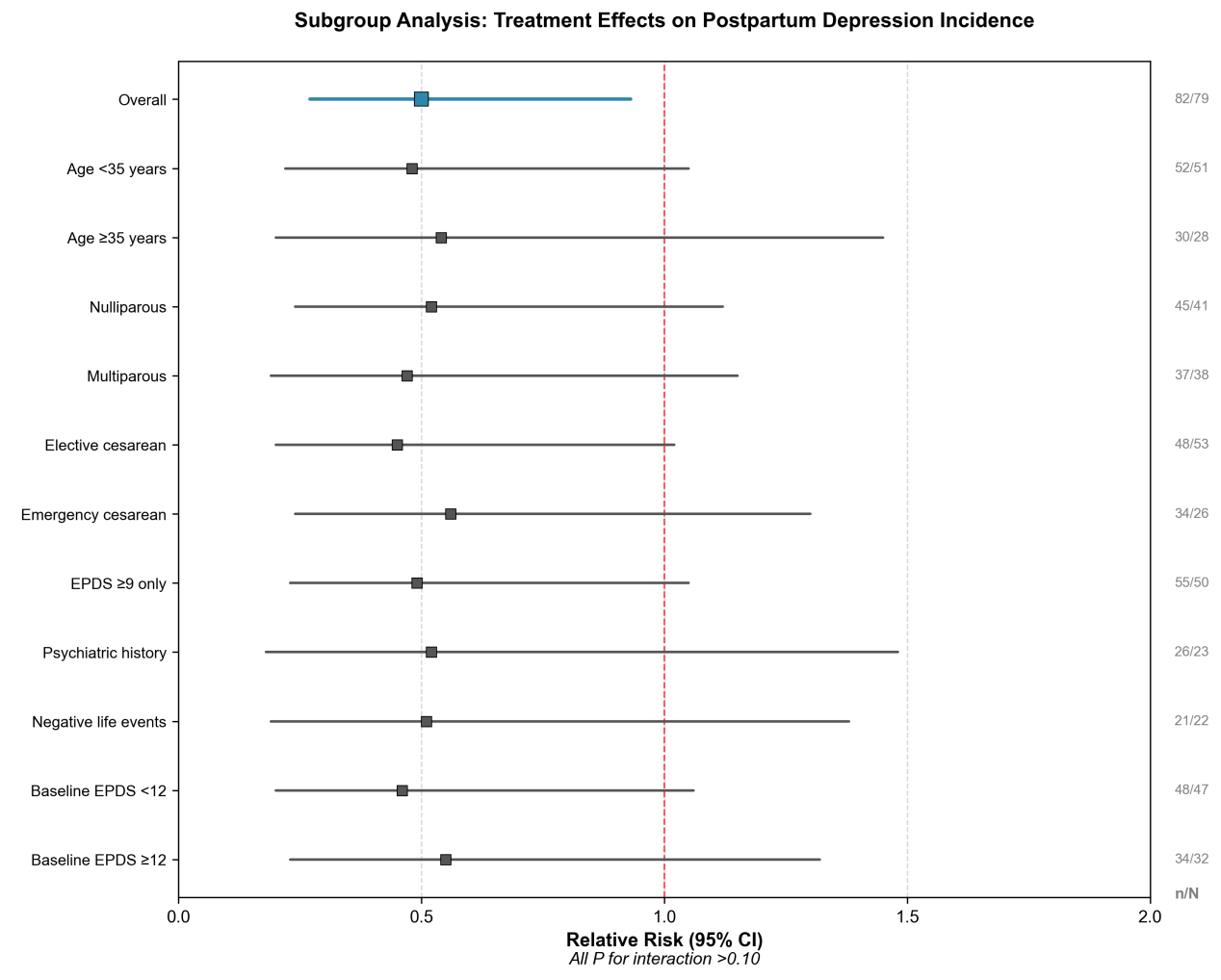


**Supplementary Figure 5**. Subgroup Analysis of Treatment Effects on Postpartum Depression Incidence

[Forest plot showing relative risks and 95% confidence intervals for PPD incidence across predefined subgroups. All subgroups favor the multimodal protocol with overlapping confidence intervals]

**Supplementary Table 1**. Correlation Analysis of Variables with Change in EPDS Score

| **Variable** | **Spearman's rho** | **P-value** | **Interpretation** |
| --- | --- | --- | --- |
| Postoperative BDNF level | -0.42 | <0.001 | Higher BDNF associated with greater EPDS reduction |
| PSQI score at day 7 | 0.51 | <0.001 | Poorer sleep quality associated with less EPDS improvement |
| Postoperative IL-6 level | 0.38 | <0.001 | Higher inflammation associated with less EPDS improvement |
| Postoperative TNF-α level | 0.29 | 0.008 | Higher inflammation associated with less EPDS improvement |
| Cumulative PCIA consumption | 0.35 | 0.001 | Higher opioid use associated with less EPDS improvement |
| NRS pain at 24h (movement) | 0.31 | 0.004 | Higher pain associated with less EPDS improvement |

BDNF = brain-derived neurotrophic factor; PSQI = Pittsburgh Sleep Quality Index; IL-6 = interleukin-6; TNF-α = tumor necrosis factor-alpha; PCIA = patient-controlled intravenous analgesia; EPDS = Edinburgh Postnatal Depression Scale; NRS = numerical rating scale. Negative correlations indicate that higher levels of the variable are associated with greater reduction (improvement) in EPDS score.
